# ‘Going the distance’: an independent cohort study of engagement and dropout among the first 100 000 referrals into a large-scale diabetes prevention program

**DOI:** 10.1136/bmjdrc-2020-001835

**Published:** 2020-12-10

**Authors:** Elizabeth Howarth, Peter J Bower, Evangelos Kontopantelis, Claudia Soiland-Reyes, Rachel Meacock, William Whittaker, Sarah Cotterill

**Affiliations:** 1 Centre for Biostatistics, School of Health Sciences, The University of Manchester, Manchester, UK; 2 Centre for Primary Care and Health Services Research, NIHR ARC Greater Manchester, School of Health Sciences, The University of Manchester, Manchester, UK; 3 Division of Informatics, Imaging and Data Sciences, The University of Manchester, Manchester, UK; 4 Centre for Primary Care and Health Services Research, School of Health Sciences, The University of Manchester, Manchester, UK; 5 Research and Innovation, Northern Care Alliance NHS Group, Salford, UK; 6 Centre for Health Economics, School of Health Sciences, The University of Manchester, Manchester, UK

**Keywords:** primary prevention, behavioral medicine, health services research, diabetes mellitus, type 2

## Abstract

**Introduction:**

Diabetes prevention programs (DPPs) are effective, in a pre-diabetic population, in reducing weight, lowering glycated hemoglobin and slowing the progression to diabetes. Little is known about the relationship between participation in DPPsand participant characteristics or service delivery. We investigated uptake and retention in England’s NHS DPP, reporting on variability among patient subgroups, providers, and sites.

**Research design and methods:**

This prospective cohort study included 99 473 adults with non-diabetic hyperglycemia referred to the English DPP between 2016 and 2017. The program seeks to change health behaviors by offering at least 16 hours of group education and exercise. Multilevel logistic regression models were used to analyze variation in uptake, retention, and completion.

**Results:**

Uptake among 99 473 adults referred to the program was 56% (55 275). Among 55 275 who started the program, 34% (18 562) achieved the required dose and 22% (12 127) completed the full course. After adjustment for variation in case mix, substantial heterogeneity in uptake and retention was seen across four service providers (uptake OR 1.77 (1.33, 2.34), 4.30 (3.01, 6.15), and 1.45 (1.07, 1.97) compared with the reference provider) and between sites (uptake for typical individuals ranged from 0.32 to 0.78 across the middle 95% of sites, intraclass correlation coefficient (ICC) 0.07). Higher levels of retention and completion were seen where some out-of-hours provision was offered (retention OR 1.32 (1.25, 1.39)).

**Conclusions:**

This study provides the first independent assessment of participation in the English DPP and the first study internationally to examine the impact of DPP service delivery on participation. When implementing a large-scale DPP, heterogeneity in service provision between different providers and sites can result in variable participation beyond that attributable to case mix, with potential consequences for effectiveness and health inequalities. Extending out-of-hours provision may improve participation in prevention programs.

**Significance of this study**

**What is already known about this subject?**

- Uptake of Diabetes Prevention Programs (DPPs), defined as the proportion enrolled on a program of those invited, can have a considerable impact on lowering diabetes risk, but until now data were not available to examine the relationship between uptake and participant characteristics or service delivery. Little is known about retention.

**What are the new findings?**

- Our paper provides the first independent assessment of participation in the English NHS DPP, using a data set large enough to examine associations with patient characteristics and service delivery.
- Variation in participation is reported by geographical site and service provider, by different methods of referral from primary care, and accounting for out-of-hours service delivery, all of which are important for understanding how variation in the delivery of a DPP intervention across a nation may impact on participation, after adjustment for variation in case mix.

**How might these results change the focus of research or clinical practice?**

- When implementing a DPP, attention is needed to promote consistency and learning across the providers and sites.
- Evidence from our study suggests that extending flexible service provision, such as out-of-hours sessions, may improve retention rates. Measures to improve uptake and retention among younger and deprived groups, and retention among minority ethnic groups, those with a disability, and people at work, may be needed.

## Introduction

Type 2 diabetes is an important and growing global health concern^1 2^ which leads to serious complications and an elevated risk of other cardiovascular diseases.^2^ Prevention of diabetes is a major international public health objective.^2 3^ Risk factors for type 2 diabetes include genetics, age, ethnicity, and obesity.^2 4^ Among those with elevated blood glucose levels, around 5%–10% are expected to develop type 2 diabetes each year, with estimates varying by country and population studied.^5^ Effective ways to delay or prevent progression to type 2 diabetes include lifestyle changes such as losing weight, eating a healthier diet, and increasing physical activity.^6–8^ Diabetes prevention programs (DPPs) have been established in many parts of the world, offering support to achieve lifestyle change among people at risk of type 2 diabetes. Evidence from trials suggests they can lead to a reduced incidence of type 2 diabetes,^9 10^ and this has been confirmed, with smaller effect sizes, in translational studies in routine practice.^11–14^

Aziz *et al*^12^ noted that the public health impact of a DPP depends on participation as well as population coverage. Their international review of 38 real-world DPPs defined uptake as the proportion enrolled on a program of those invited, categorized as low (≤33%), moderate (34%–66%), or high (≥67%). They found wide variation in uptake and concluded that uptake had a considerable impact on lowering diabetes risk. Retention of participants in DPPs is less often reported, and definitions used to measure uptake, retention, and completion vary between studies. Variation in participation may reflect different recruitment strategies and method of enrollment, assorted retention or completion milestones, as well as diversity in intensity and duration. This prevents straightforward comparisons between interventions. Poor uptake and retention are likely to result in wasted resources. Analysis of variation in DPP participation across demographic groups has not been possible in earlier studies because large population samples are needed; such analyses could reveal the extent of any inequalities in access, allowing providers to target resources to areas or groups where participation is low.

The Healthier You: NHS DPP is a behavior change program in England offering education on healthy eating and lifestyle, and help to lose weight and increase physical exercise,^15^ designed based on international evidence and expert opinion.^11 16^ Between 2016 and 2019 the DPP was introduced across England in three waves, delivered by four private service providers.^17^ The National Health Service (NHS) in England is organized geographically into clinical commissioning groups (CCGs), and at a higher level into sustainability and transformation partnerships (STPs). In general, a single provider was commissioned by each STP, but local implementation of referrals from primary care was managed by CCGs.

The DPP targeted adults over 18 with non-diabetic hyperglycemia (NDH), defined by the National Institute for Health and Care Excellence as glycated hemoglobin (HbA1c) of 6.0%–6.4% (42–47 mmol/mol) or fasting plasma glucose (FPG) level of 5.5–6.9 mmol/L. Eligible patients were identified in primary care and referred to a local provider via one of two main routes. ‘Consultation route’ referrals were made by a primary care professional following a consultation with the patient. ‘Letter route’ referrals resulted after a letter was sent to eligible patients identified by a search of general practice records, informing them that they were at elevated risk of type 2 diabetes and advising them to contact their local provider to self-refer.

Participants were offered an individual initial assessment, followed by regular group education and exercise sessions, comprising at least 16 hours of contact over 9–12 months.

We aimed to investigate the extent of uptake, retention and completion in the NHS DPP and report on variation in service delivery and participation among patient subgroups and between different providers and sites.

The team involved in developing and implementing the DPP has already published results on program participation^18 19^ using the same data source. For the first time, variation in participation is reported by geographical site and service provider, by different methods of referral from primary care, and accounting for out-of-hours service delivery, all of which are important for understanding how variation in the delivery of a DPP intervention across a nation may impact on participation, after adjustment for variation in case mix.

## Research design and methods

### Data

This study used individual-level patient data collected by service providers on all referrals received from April 2016 to October 2017. Phased service implementation meant that some sites were not represented in the data, while some had more established services than others. Demographic and attendance data, and health and well-being measures were collected over the course of participants’ engagement. Those who declined a referral at consultation were not represented in the data, nor were those sent a letter who did not subsequently contact a provider.

#### Patient characteristics

Gender and age were recorded at referral. The 2015 Index of Multiple Deprivation score, categorized using English population quintiles, was obtained as a measure of local area deprivation using the patient’s postcode. Ethnicity, employment status, disability, and smoking were recorded for those who attended an initial assessment.

#### Patient health measures

HbA1c or FPG measured in primary care was recorded at referral. HbA1c or FPG was measured again by providers at initial assessment for those whose referral measure was over 3 months old, and at 6 months and in the final session for participants retained to those milestones. Weight was measured at initial assessment and at each intervention session but was not routinely recorded at referral. Well-being was measured at initial assessment and final session using the Warwick Edinburgh Mental Wellbeing Scale (WEMWBS).

#### Service delivery

Provider, site (STP, CCG), and date and source of referral were recorded for all referrals. ‘Service maturity’ is the number of months from service establishment at that site to referral date. Times of sessions were recorded for initial assessment and any subsequent intervention sessions. ‘Out-of-hours’ provision describes any session starting before 09:00 or after 17:00.

### Outcome measures

We used three outcome measures of participation in the DPP: uptake, retention, and completion (table 1). Uptake was defined as attendance at initial assessment or at least one intervention session and was analyzed with respect to all recorded referrals (‘referrals’ n=99 473). Retention to 60% attendance aligns with the NHS DPP delivery team’s definition of completion.^19^ We additionally used a stricter measure of completion, defined as attendance at the final intervention session or the recording of some final measure of HbA1c, FPG, or WEMWBS, plus at least 60% attendance. We examined variation in the proportions achieving each milestone against patient and service characteristics, and across sites, accounting for both STP and CCG level. Analyses of retention and completion were restricted to those who started the program (‘attenders’ n=55 275).

**Table 1:** Definitions of participation outcomes

Milestone (outcome measure)	Definition	Analysis cohort
Uptake	Attendance at initial assessment or at least one intervention session.	All recorded referrals (‘referrals’ n=99 473).
Retention to 60% (DPP completion)	Attended at least 60% of sessions (aligns with the DPP delivery team’s definition of completion).^19^	Those who started the program, attending at least once (‘attenders’ n=55 275).
Completion	Attendance at the final intervention session or the recording of some final health measure (HbA1c or well-being), plus at least 60% attendance.	Those who started the program, attending at least once (‘attenders’ n=55 275).

DPP, diabetes prevention program; HbA1c, glycated hemoglobin.

### Analysis

Logistic regression models were used to estimate adjusted associations of gender, age, deprivation, HbA1c, provider, and referral source with each participation milestone, and additionally of disability, employment status, smoking, weight, WEMWBS score, and out-of-hours provision (all unavailable for non-attenders) with retention and completion measures. The largest provider (A) was chosen as the reference provider. Causal relationships were considered in order to adjust for measured confounders, while avoiding adjustment for intermediaries or common descendants.^20 21^ Details are given with results. Stata V.15 was used in all analyses.

Where site was identified as a potential intermediary between a characteristic and outcome (eg, ethnicity or age may influence where an individual lives), fixed effects logistic regression was used with robust estimation to allow for clustering by CCG, providing estimates of marginal associations across sites. Otherwise, mixed effects models with nested independent random intercept terms for CCG within STP were used to allow for clustering. Variation across sites was reported using 95% coverage intervals, the range of participation rates within which we would expect 95% of sites to lie for typical individuals (white, 65 years old, retired, non-smoking woman in the middle deprivation group, reporting no disability, with median values of HbA1c, weight, and WEMWBS). The coverage interval was estimated from the output of the multilevel model and represents the upper and lower bounds for site participation rates after controlling for other variables in the model (see online supplemental material).^22^

10.1136/bmjdrc-2020-001835.supp1 Supplementary data

Multiple imputation (M=10) by fully conditional specification (FCS) was used to reduce bias due to missingness of up to 30% among covariates.^23 24^ Imputation was performed separately for non-attenders and attenders, since variables collected at initial assessment were plausibly missing not at random among non-attenders (ie, differently distributed among non-attenders compared with attenders, conditional on observed data).^25^ Therefore, these were neither imputed nor included in the analysis of uptake among all referrals, but were added to imputation and analysis models for retention and completion among attenders.^26^ Participation outcomes were completely observed (uptake among all referrals; retention and completion among attenders) and were included in imputation models. This approach ensured that for each analysis all relevant covariates, outcomes and auxiliary variables were included in the FCS for the corresponding subsample (see online supplemental material).^27^

## Results

### Patient characteristics

Descriptive summaries of patient characteristics are reported in table 2. Among referrals, the proportion of women was 55% and the median age was 64 (IQR 19) years, with 53% aged between 60 and 79 years. Proportions were close to 20% in each of the five deprivation strata but with slightly higher proportions from more deprived compared with less deprived areas. Among attenders, 75%, 13%, 8%, and 4% reported belonging to white, Asian, black, and other ethnicities, respectively. A majority (57%) were retired, 19% reported disability, and 91% were non-smokers.

**Table 2:** Patient characteristics overall and by provider*

	All referrals					Attenders				
	Overall	Provider				Overall	Provider			
		A	B	C	D		A	B	C	D
n (row %)	99 473	39 690 (39.9)	10 657 (10.7)	20 944 (21.1)	28 182 (28.3)	55 275	23 586 (42.7)	6667 (12.1)	13 882 (25.1)	11 140 (20.2)
Gender, n (%)										
Male	44 793 (45.4)	17 578 (44.6)	4456 (43.7)	9281 (44.5)	13 478 (47.8)	24 577 (44.7)	10 275 (43.8)	2850 (43.1)	6206 (45.0)	5246 (47.1)
Female	53 835 (54.6)	21 843 (55.4)	5739 (56.3)	11 549 (55.4)	14 704 (52.2)	30 404 (55.3)	13 169 (56.2)	3757 (56.9)	7584 (55.0)	5894 (52.9)
Age (years)										
Median (IQR)	64 (19)	65 (18)	65 (19)	62 (19)	65 (19)	66 (17)	66 (16)	67 (16)	64 (18)	67 (17)
Age group, n (%)										
<40	4923 (5.0)	1716 (4.3)	536 (5.0)	1319 (6.3)	1352 (4.8)	1930 (3.5)	755 (3.2)	191 (2.9)	647 (4.7)	337 (3.0)
40–49	11 539 (11.6)	4228 (10.7)	1270 (11.9)	2873 (13.7)	3168 (11.2)	5175 (9.4)	2006 (8.5)	602 (9.0)	1621 (11.7)	946 (8.5)
50–59	21 199 (21.3)	8321 (21.0)	2139 (20.1)	4811 (23.0)	5928 (21.0)	10 957 (19.8)	4622 (19.6)	1176 (17.6)	3063 (22.1)	2096 (18.8)
60–69	27 560 (27.7)	11 372 (28.7)	2926 (27.5)	5515 (26.4)	7747 (27.5)	16 585 (30.0)	7325 (31.1)	2016 (30.2)	3903 (28.2)	3341 (30.0)
70–79	24 878 (25.0)	10 183 (25.7)	2817 (26.4)	4763 (22.8)	7115 (25.3)	15 576 (28.2)	6583 (27.9)	2054 (30.8)	3550 (25.6)	3389 (30.4)
80+	9345 (9.4)	3870 (9.8)	967 (9.1)	1639 (7.8)	2869 (10.2)	5036 (9.1)	2295 (9.7)	628 (9.4)	1082 (7.8)	1031 (9.3)
Deprivation, n (%)										
1 (most deprived)	21 054 (21.2)	7065 (17.8)	4108 (38.8)	5403 (26.1)	4478 (15.9)	10 856 (19.7)	4028 (17.1)	2092 (31.6)	3219 (23.4)	1517 (13.6)
2	21 291 (21.5)	8753 (22.1)	2330 (22.0)	5136 (24.8)	5072 (18.0)	11 305 (20.5)	4881 (20.7)	1480 (22.3)	3174 (23.1)	1770 (15.9)
3	20 582 (20.8)	9775 (24.6)	1854 (17.5)	3831 (18.5)	5122 (18.2)	11 899 (21.6)	5790 (24.6)	1344 (20.3)	2737 (19.9)	2028 (18.2)
4	18 605 (18.8)	7919 (20.0)	1172 (11.1)	3309 (16.0)	6205 (22.0)	10 730 (19.5)	4816 (20.4)	866 (13.1)	2388 (17.4)	2660 (23.9)
5 (least deprived)	17 641 (17.8)	6173 (15.6)	1115 (10.5)	3061 (14.8)	7292 (25.9)	10 319 (18.7)	4068 (17.3)	845 (12.8)	2245 (16.3)	3161 (28.4)
Ethnicity†, n (%) (available for attenders only)										
White						38 439 (74.6)	17 206 (74.6)	5303 (81.9)	7694 (67.3)	8236 (78.3)
Asian						6804 (13.2)	3115 (13.5)	736 (11.4)	1550 (13.6)	1403 (13.3)
Black						4298 (8.3)	1717 (7.4)	357 (5.5)	1702 (14.9)	522 (5.0)
Other						1967 (3.8)	1036 (4.5)	77 (1.2)	493 (4.3)	361 (3.4)
Employment, n (%) (available for attenders only)										
Retired						23 869 (57.3)	12 932 (56.2)	3577 (60.2)	4011 (57.4)	3349 (58.4)
Other						4542 (10.9)	2559 (11.1)	676 (11.4)	712 (10.2)	595 (10.4)
Disability, n (%) (available for attenders only)										
No						38 700 (81.1)	18 504 (80.7)	4160 (75.5)	7742 (87.5)	8294 (79.5)
Yes						9020 (18.9)	4421 (19.3)	1350 (24.5)	1108 (12.5)	2141 (20.5)
Smoking, n (%) (available for attenders only)										
Smoker						3620 (8.3)	2129 (9.2)	499 (8.8)	321 (5.5)	671 (7.6)
Ex-smoker						122 (0.3)	0 (0.0)	120 (2.1)	2 (0.0)	0 (0.0)
Non-smoker						39 701 (91.4)	20 955 (90.8)	5058 (89.1)	5504 (94.5)	8184 (92.4)
Referral HbA1c, % (mmol/mol)										
n Median (IQR)	82 418 6.1 (43) (0.3 (3))	36 870 6.1 (43) (0.3 (3))	10 133 6.1 (43) (0.3 (3))	19 520 6.1 (43) (0.3 (3))	15 895 6.2 (44) (0.3 (3))	49 088 6.1 (43) (0.3 (3))	21 658 6.1 (43) (0.3 (3))	6345 6.1 (43) (0.3 (3))	13 130 6.1 (43) (0.3 (3))	7955 6.2 (44) (0.2 (2))
Referral FPG (mmol/L)										
n Median (IQR)	12 840 5.8 (0.6)	9907 5.7 (0.8)	556 6.1 (0.6)	796 5.9 (0.6)	1581 5.9 (0.5)	6845 5.8 (0.6)	5210 5.7 (0.7)	343 6 (0.6)	573 5.9 (0.6)	719 6 (0.5)
Referral blood glucose category‡, n (%)										
Normal	1735 (2.0)	503 (1.3)	196 (1.8)	976 (4.8)	60 (0.3)	684 (1.3)	216 (0.9)	55 (0.8)	395 (2.9)	18 (0.2)
NDH	85 803 (97.5)	38 955 (98.3)	10 396 (97.7)	19 083 (94.3)	17 369 (99.4)	51 695 (98.4)	23 257 (98.8)	6591 (99.0)	13 200 (96.8)	8647 (99.8)
Type 2 diabetes	452 (0.5)	174 (0.4)	53 (0.5)	184 (0.9)	41 (0.2)	134 (0.3)	70 (0.3)	12 (0.2)	48 (0.4)	4 (0.1)
Initial assessment weight (kg) (available for attenders only)										
n Median (IQR)						46 240 81.8 (24.2)	22 378 81.0 (23.8)	6634 82.0 (23.8)	6383 81.8 (23.9)	10 845 83.3 (24.5)
Initial assessment BMI category§, n (%) (available for attenders only)										
Underweight/healthy weight						7665 (16.8)	3847 (17.2)	1068 (16.1)	1189 (19.2)	1561 (14.9)
Overweight						16 742 (36.7)	8205 (36.7)	2465 (37.2)	2326 (37.6)	3746 (35.8)
Obese						21 253 (46.6)	10 322 (46.1)	3101 (46.7)	2673 (43.2)	5157 (49.3)
WEMWBS score (available for attenders only)										
n Median (IQR)						40 446 54 (13)	16 403 54 (12)	5994 54 (13)	10 914 54 (15)	7135 52 (14)

*Levels of missing data can be inferred, or see online supplemental table S4 for details.

†‘Asian’ comprises those reporting Indian, Pakistani, Bangladeshi, Chinese, or ‘other Asian’ ethnicity; ‘black’ comprises those reporting Caribbean, African, or ‘other black’ ethnicity; ‘other’ comprises those reporting any other non-white ethnicity, including ‘mixed’ groups.

‡HbA1c and FPG groups combined; ‘normal’ range: HbA1c <6.0% (42 mmol/mol) or FPG <5.5 mmol/L; ‘NDH’ range: HbA1c 6.0%–6.4% (42–47 mmol/mol) or FPG 5.5–6.9 mmol/L inclusive; ‘type 2 diabetes’ range: HbA1c >6.4% (47 mmol/mol) or FPG >6.9 mmol/L.

§‘Underweight’: <18.5 kg/m^2^; ‘healthy weight’: 18.5–24.9 kg/m^2^; ‘overweight’: 25–29.9 kg/m^2^; and ‘obese’: 30 kg/m^2^ or higher.

BMI, body mass index; FPG, fasting plasma glucose; HbA1c, glycated hemoglobin; NDH, non-diabetic hyperglycemia; WEMWBS, Warwick Edinburgh Mental Wellbeing Scale.

HbA1c at referral was skewed with a majority toward the lower end of the eligible range of values, with a median of 6.1% (43 mmol/mol) (IQR 0.3% (3 mmol/mol)). At initial assessment (among attenders) the median weight was 81.8 kg (IQR 24.2); in terms of body mass index, 17% were underweight or of healthy weight, 37% overweight, and 47% obese. Considerable variation in case mix was seen across providers with respect to deprivation, ethnicity, and disability, less so for other characteristics (table 2).

### Service features

Descriptive summaries of service features are given in online supplemental table S1. The majority (71%) of the received referrals were made via consultation, with 28% via letter. Some out-of-hours delivery was offered to 16% of attenders; there was heterogeneity across providers in both referral source and out-of-hours provision.

### Participation outcomes

**Figure 1:**
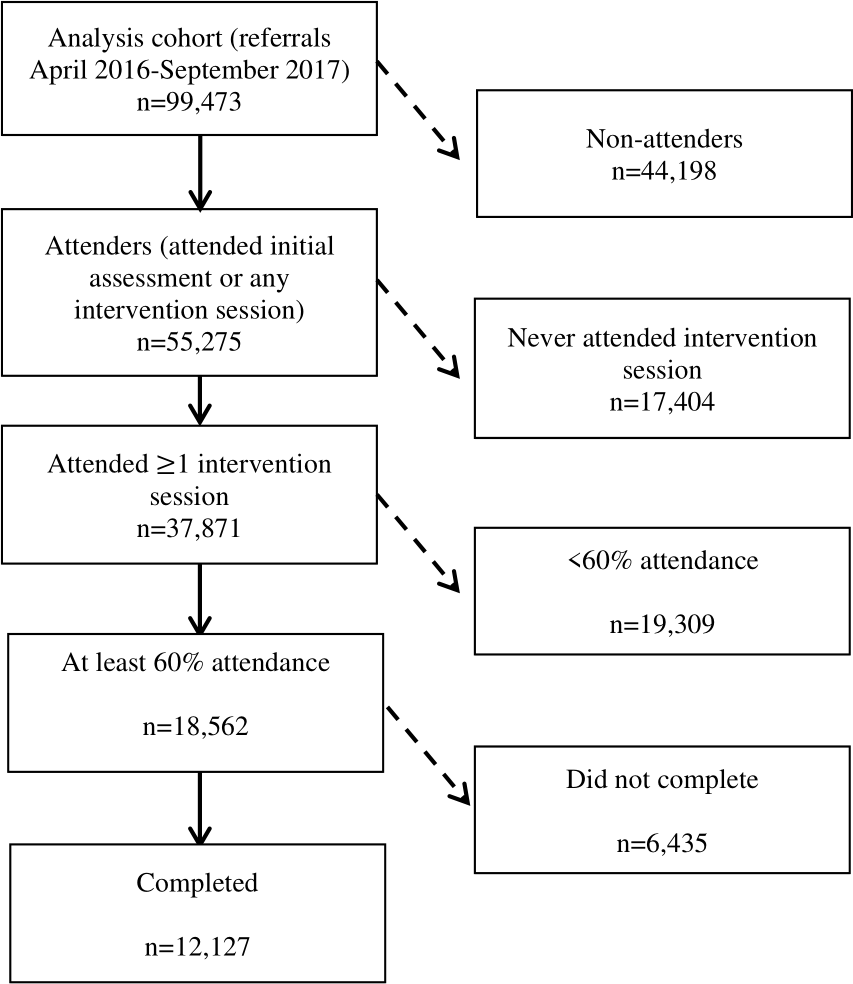
Participant numbers.

Descriptive summaries are available in online supplemental tables S2 and S3, and the relative proportions reaching each outcome are shown in figure 1. Outcomes are defined in table 1.

### Uptake

Overall 55 275 out of 99 473 recorded referrals (56%) took up a place. Uptake was slightly higher among women than men (OR 1.08, 95% CI (1.04 to 1.11)) and increased with age (OR 1.17 (1.15 to 1.20) per 5 years) among those aged up to 70, decreasing with older age (table 3). Uptake was lowest among those living in the most deprived areas, and increased with affluence (OR 0.65 (0.61 to 0.68) for the most compared with the least deprived group). HbA1c at referral was not associated with uptake (OR 0.99 (0.98 to 1.00) per 0.1% (1 mmol/mol)).

**Table 3:** Associations of patient and service characteristics with uptake

	OR	SE	95% CI
Gender*			
Female	1.08	0.02	1.04 to 1.11
Male (reference)	1		
Age* <70 (per 5 years)	1.17	0.01	1.15 to 1.20
Age* 70+ (per 5 years)	0.86	0.01	0.83 to 0.88
Deprivation†‡			
1 (most deprived)	0.65	0.02	0.61 to 0.68
2	0.66	0.02	0.63 to 0.70
3	0.78	0.02	0.74 to 0.82
4	0.84	0.02	0.81 to 0.88
5 (least deprived) (reference)	1		
Referral HbA1c (per 0.1% (1 mmol/mol))§‡	0.99	0.005	0.98 to 1.00
Provider¶‡			
A	1.77	0.25	1.33 to 2.34
B	4.30	0.78	3.01 to 6.15
C	1.45	0.23	1.07 to 1.97
D (reference)	1		
Referral source¶‡			
Letter	4.33	0.11	4.13 to 4.55
Consultation (reference)	1		
${\hat{\sigma }}_{STP}^{2}+{\hat{\sigma }}_{CCG}^{2}$(log odds scale)	0.26		
Intraclass Correlation Coefficient (ICC) (CCG level)	0.07		

*Associations of gender and age with uptake were mutually adjusted and marginal over site (no random terms), representing the estimated means across the cohort.

†Adjusted for gender and age.

‡Random intercept terms for STP and CCG included; associations are conditional, representing the estimated individual-level mean association after random variation between sites has been accounted for.

§Adjusted for gender, age and deprivation.

¶Associations of service characteristics with uptake were mutually adjusted and adjusted for gender, age, deprivation, HbA1c at referral and service maturity (months from service establishment to referral date).

CCG, clinical commissioning group; HbA1c, glycated hemoglobin; STP, sustainability and transformation partnership.

Uptake was much higher among referrals via letter compared with consultation (OR 4.33 (4.13 to 4.55)). After adjustment for case mix, referral source, and service maturity, there remained large variation in uptake across providers (OR 1.77 (1.33 to 2.34), 4.30 (3.01 to 6.15), and 1.45 (1.07 to 1.97) compared with the reference provider) (table 3). Furthermore, after adjustment for case mix, there was considerable residual variation across sites: the uptake rates for typical individuals lie between 0.32 and 0.78 across the middle 95% of sites (intraclass correlation coefficient (ICC) was 0.07 at the CCG level).

**Figure 2:**
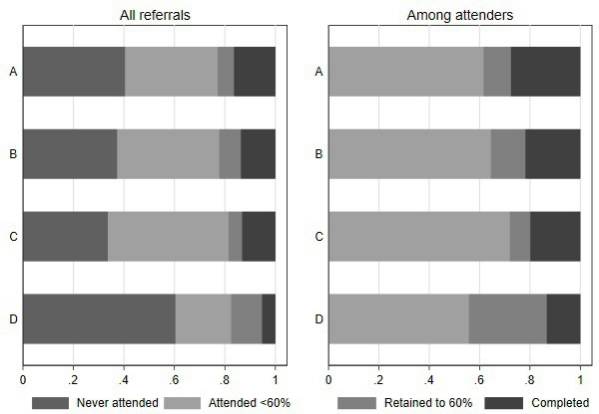
Relative proportions reaching participation milestones, by provider.

### Retention and completion among attenders

Analyses of retention and completion were restricted to those who started the program (n=55 275). Of these, 17 404 (31%) attended initial assessment only and 37 871 (69%) went on to attend at least one intervention session (figure 1); the median attendance was 31% of sessions. Overall 18 562 attenders (34%) reached the 60% attendance milestone used as the threshold for our definition of retention, with women slightly more likely to do so than men (OR 1.06 (1.01 to 1.11)) (table 4). Retention increased with age (OR 1.27 (1.25 to 1.29) per 5 years) among those aged up to 70, decreasing beyond this point. Retention to 60% was less common among all minority ethnicities (Asian (OR 0.75 (0.63 to 0.91)), black (OR 0.83 (0.71 to 0.99)), and other ethnicities (OR 0.74 (0.63 to 0.86))) compared with white groups, and increased with affluence (OR 0.65 (0.60 to 0.70) for the most compared with the least deprived group). Lower retention was seen among those reporting a disability, those in employment, or ‘other’ occupational groups compared with retirees and ex-smokers or current smokers (table 4). Neither weight nor HbA1c at initial assessment was associated with retention.

**Table 4:** Associations of patient and service characteristics with retention and completion among attenders

	60% attendance			Completion		
	OR	SE	95% CI	OR	SE	95% CI
Gender*						
Female	1.06	0.02	1.01 to 1.11	1.06	0.03	1.01 to 1.11
Male (reference)	1			1		
Age* <70 (per 5 years)	1.27	0.01	1.25 to 1.29	1.30	0.01	1.27 to 1.32
Age* 70+ (per 5 years)	0.81	0.01	0.79 to 0.84	0.82	0.01	0.79 to 0.84
Ethnicity†						
Asian	0.75	0.07	0.63 to 0.91	0.78	0.10	0.61 to 1.01
Black	0.83	0.07	0.71 to 0.99	0.85	0.08	0.70 to 1.03
Other	0.74	0.06	0.63 to 0.86	0.76	0.07	0.64 to 0.90
White (reference)	1			1		
Deprivation						
1 (most deprived)	0.65	0.02	0.60 to 0.70	0.68	0.03	0.62 to 0.74
2	0.74	0.02	0.69 to 0.79	0.79	0.03	0.73 to 0.84
3	0.83	0.03	0.78 to 0.88	0.87	0.03	0.81 to 0.93
4	0.89	0.03	0.84 to 0.94	0.89	0.03	0.83 to 0.95
5 (least deprived) (reference)	1			1		
Disability‡§						
Yes	0.81	0.02	0.77 to 0.86	0.82	0.03	0.77 to 0.88
No (reference)	1			1		
Employment‡§						
Employed	0.74	0.03	0.69 to 0.80	0.69	0.03	0.63 to 0.76
Retired (reference)	1			1		
Other	0.81	0.05	0.72 to 0.91	0.82	0.06	0.71 to 0.94
Smoking‡§						
Ex-smoker or current smoker	0.52	0.02	0.48 to 0.57	0.56	0.03	0.50 to 0.62
Non-smoker (reference)	1			1		
Initial assessment weight (per 5 kg)¶§						
Up to 65 kg	1.02	0.02	0.99 to 1.05	1.01	0.02	0.97 to 1.05
65–140 kg	0.99	0.004	0.98 to 0.99	0.98	0.004	0.97 to 0.99
Over 140 kg	0.99	0.03	0.93 to 1.05	1.04	0.04	0.97 to 1.11
Initial assessment HbA1c (per 0.1% (1 mmol/mol))§¶	1.01	0.01	0.99 to 1.02	1.02	0.01	1.00 to 1.04
Initial assessment WEMWBS (per 5 points)¶§						
Up to 55 points	1.09	0.01	1.07 to 1.10	1.09	0.01	1.07 to 1.11
Over 55 points	0.93	0.01	0.91 to 0.96	0.93	0.01	0.90 to 0.96
Provider**§						
A	1.38	0.13	1.15 to 1.66	1.57	0.30	1.09 to 2.28
B	1.86	0.21	1.49 to 2.32	1.43	0.31	0.95 to 2.18
C	0.74	0.08	0.60 to 0.91	1.08	0.20	0.75 to 1.54
D (reference)	1			1		
Referral source**§						
Letter	1.04	0.03	0.98 to 1.10	1.04	0.04	0.97 to 1.11
Consultation (reference)	1					
Out-of-hours provision**§						
Some	1.32	0.04	1.25 to 1.39	1.28	0.04	1.21 to 1.37
None (reference)	1			1		
${\hat{\sigma }}_{STP}^{2}+{\hat{\sigma }}_{CCG}^{2}$(log odds scale)	0.08			0.33		
Intraclass Correlation Coefficient (ICC) (CCG level)	0.03			0.09		

*Associations of gender, age and ethnicity with retention and completion were mutually adjusted and marginal over site (no random terms), representing the estimated means across the attenders subgroup.

†‘Asian’ comprises those reporting Indian, Pakistani, Bangladeshi, Chinese or ‘other Asian’ ethnicity; ‘black’ comprises those reporting Caribbean, African or ‘other black’ ethnicity; ‘other’ comprises those reporting any other non-white ethnicity, including ‘mixed’ groups.

‡Associations of disability, employment, deprivation and smoking with retention and completion were mutually adjusted and adjusted for gender, age and ethnicity.

§Random intercept terms for STP and CCG included; associations are conditional, representing the estimated individual-level mean association after random variation between sites has been accounted for.

¶Associations of weight, HbA1c and WEMWBS score with retention and completion were mutually adjusted and adjusted for gender, age, ethnicity, employment, disability, deprivation and smoking.

**Associations of service characteristics with retention and completion were mutually adjusted and adjusted for gender, age, ethnicity, deprivation, smoking, weight at initial assessment, HbA1c at initial assessment, WEMWBS score at initial assessment and service maturity.

CCG, clinical commissioning group; HbA1c, glycated hemoglobin; STP, sustainability and transformation partnership; WEMWBS, Warwick Edinburgh Mental Wellbeing Scale.

As for uptake, there remained considerable variation in retention across providers after adjustment for case mix and service features (OR 1.38 (1.15 to 1.66), 1.86 (1.49 to 2.32), and 0.74 (0.60 to 0.91) compared with the reference provider) (figure 2). Provision of out-of-hours delivery was associated with improved retention (OR 1.32 (1.25 to 1.39)) and, unlike uptake, retention was similar regardless of referral source. There was considerable residual variation across sites, although less than for uptake, due in part to the larger available set of explanatory factors. After adjustment for case mix, there was considerable residual variation across sites: the 60% retention rates for typical individuals lie between 0.32 and 0.78 across the middle 95% of sites (ICC was 0.03 at the CCG level).

Overall 12 127 out of 55 275 attenders completed the program (22%), with similar associations to those reported for retention (table 4). Again, large variation across providers was seen after adjustment for case mix and service features, although provider rank order was different, and again there was large residual variation across sites.

## Conclusions

There is extensive evidence that lifestyle changes among people with NDH, such as losing weight, eating a healthier diet and increasing physical activity, can delay or prevent progression to type 2 diabetes,^6–8^ and DPPs which offer support to achieve lifestyle change can be effective in reducing risk.^11–14^ Participation in such programs varies widely between settings,^12^ although direct comparisons are difficult due to variation in terminology. Low uptake and retention rates result in wasted resources and are associated with reduced effectiveness;^12^ variation in participation across an eligible population may reflect variation in opportunity of access and may exacerbate existing health inequalities.

Among all referrals received by the DPP between April 2016 and September 2017 inclusive, 56% took up a place on the program, although this does not account for an unknown number of people offered a referral or invitation to self-refer from primary care who chose not to pursue the opportunity. Of those who took up a place, 34% went on to attend the proportion of sessions recommended by the DPP delivery team and 22% completed the program. The median attendance among participants was a third of the course. Uptake of the NHS DPP compared well with other DPPs internationally and would be classed as ‘moderate’ according to the classification by Aziz *et al.*^12^

The highest levels of uptake were seen among women around the age of 70 living in the least deprived areas. The same characteristics were associated with the highest proportions reaching 60% attendance and completion, where those with the highest levels were additionally white, retired, reported no disabilities, and identified as non-smokers. With the exception of low deprivation, these were typical (modal) characteristics among all recorded referrals. One possible interpretation of this is that the DPP has been most successful at engaging and retaining majority groups from among those referred, although efforts were made in some cases to tailor DPP provision to fit the cultural needs of local communities.^28^ Another interpretation is that those referred tend to represent those who participate best, and both of these may be true simultaneously.

Measures may be needed to improve uptake and retention among those groups less likely to participate, or to identify other more acceptable interventions, if the disparities are to be redressed. Targets to refer and retain proportionally more participants from minority ethnic groups and deprived areas have already been implemented by NHS England, based on their own analyses,^18^ as part of commissioning changes made in 2019, using payment incentives for providers. It remains to be seen whether this will lead to improvements in uptake and retention among these groups.

The DPP was successful in attracting and retaining participants across the eligible range of HbA1c and across the spectrum of weight; it does not appear that people were more or less likely to engage based on their risk, although there is some evidence that the risk of diabetes is not well understood in the at-risk population.^29^

Considerable variation in achievement of all three participation milestones was found between providers and across sites after adjustment for all available factors, suggesting that there may be scope for learning by providers and sites with low participation from those with better levels. Some flexibility in local provision was encouraged in the English DPP and may be desirable to tailor the content to local circumstances or to different populations,^28^ but variation in participation may be an unintended consequence. Higher levels of retention and completion were seen where some out-of-hours provision was offered, suggesting that extending out-of-hours provision may improve participation in the DPP.

There are strengths and limitations to this study. This is the first study, to our knowledge, reporting variation in participation by service characteristics, such as geographical site and service provider, method of referral from primary care, and out-of-hours service delivery, illustrating the importance of addressing variation in delivery within a single DPP. Ours is the first independent assessment of participation in the English DPP; the results are broadly consistent with those reported by the delivery team,^18 19^ given differences in methodology. Additional results, unique to this paper, include the relationship between DPP participation and disability, employment status, smoking, baseline weight, and blood glucose level. We have reported on completion of the program as well as uptake and 60% attendance. Multilevel modeling with robust estimation, accounting for plausible causal relationships, use of linear splines to model non-linear relationships and multiple imputation all add rigor to the analysis.

Selection into the cohort was dependent on various factors, including self-selection, especially among those referred via letter.^29^ It is unknown how many candidates declined a referral at consultation or failed to self-refer after receiving an invitation by letter. Results apply to those who accept the initial invitation to have a referral recorded; they do not represent eligible candidates who declined at this early stage. Data on ethnicity, disability, employment, and smoking were unavailable for analysis of uptake and, as with all observational studies, additional unmeasured sources of bias may remain unaccounted for. As with any use of parametric modeling, disparities between model assumptions and reality may result in residual confounding.

In conclusion, over half of those accepting a referral from primary care to the NHS DPP took up a place, and a third of those who started went on to attend the proportion of sessions recommended by the DPP delivery team. The DPP has been able to retain people regardless of differences in baseline weight and blood glucose. There was substantial heterogeneity between providers and sites in their uptake and retention rates, suggesting the need for greater consistency and learning across the providers and sites. Extending flexible service provision such as out-of-hours sessions can improve retention rates. Measures to improve uptake and retention among younger people and deprived groups, and retention among minority ethnic groups, those with a disability, and people at work, may be needed.
