# Supplementary material for: ‘Going the distance’: an independent cohort study of engagement and dropout among the first 100 000 referrals into a large-scale diabetes prevention program
Source: BMJ Open Diabetes Res Care. 2020 Dec 10;8(2):e001835. doi: 10.1136/bmjdrc-2020-001835 (PMC7733095; doi:10.1136/bmjdrc-2020-001835)
Supplement: Supplementary data [file bmjdrc-2020-001835supp001.pdf]

Online-Only Supplemental Material

Supplemental Table S1: Service characteristics overall and by provider

	**full cohort**							**attenders**					
	overall		provider					overall		provider			
			A	B	C	D				A	B	C	D
n<br>(row %)	99,473		39,690<br>(39·9)	10,657<br>(10·7)	20,944<br>(21·1)	28,182<br>(28·3)		55,275		23,586<br>(42·7)	6,667<br>(12·1)	13,882<br>(25·1)	11,140<br>(20·2)
**Referral source n (%)**													
consultation (GP/ health check)	71,109<br>(71·5)		26,568<br>(66·9)	9,877<br>(92·7)	6,482<br>(31·0)	28,182<br>(100·0)		33,232<br>(60·1)		12,580<br>(53·3)	6,092<br>(91·4)	3,420<br>(24·6)	11,140<br>(100·0)
letter (self-referral after advice)	28,318<br>(28·5)		13,112<br>(33·0)	767<br>(7·2)	14,439<br>(68·9)	0<br>(0·0)		22,020<br>(39·8)		11,002<br>(46·7)	569<br>(8·5)	10,449<br>(75·3)	0<br>(0·0)
other sources	46<br>(0·0)		10<br>(0·0)	13<br>(0·1)	23<br>(0·1)	0<br>(0·0)		23<br>(0·1)		4<br>(0·0)	6<br>(0·1)	13<br>(0·1)	0<br>(0·0)
**Out-of-hours provision n (%)** (available for attenders only)													
none								46,400<br>(83·9)		20,802<br>(88·2)	6,101<br>(91·5)	12,429<br>(89·5)	7,068<br>(63·4)
some								8,875<br>(16·1)		2,784<br>(11·8)	566<br>(8·5)	1,453<br>(10·5)	4,072<br>(36·6)

Supplemental Table S2: Participation by patient characteristics - descriptive summary[a]

	**uptake full cohort n=99,473**		**60% attendance attenders n=55,275**		**completion attenders n=55,275**	
	No	yes	no	yes	no	yes
**Overall n (%)**	44,198 (44·4)	55,275 (55·6)	36,713 (66·4)	18,562 (33·6)	43,148 (78·1)	12,127 (21·9)
**Gender n (%)**						
male	20,216 (45·1)	24,577 (54·9)	16,426 (66·8)	8,151 (33·2)	19,256 (78·3)	5,321 (21·7)
female	23,431 (43·5)	30,404 (56·5)	20,078 (66·0)	10,326 (34·0)	23,646 (77·8)	6,758 (22·2)
**Age (years)**						
n	44,185	55,259	36,697	18,562	43,132	12,127
median	62	66	64	68	65	69
(IQR)	(21)	(17)	(19)	(13)	(18)	(12)
**Age group n (%)**						
<40	2,993 (60·8)	1,930 (39·2)	1,707 (88·4)	223 (11·6)	1,797 (93·1)	133 (6·9)
40-49	6,364 (55·2)	5,175 (44·9)	4,220 (81·5)	955 (18·5)	4,654 (89·9)	521 (10·1)
50-59	10,242 (48·3)	10,957 (51·7)	8,000 (73·0)	2,957 (27·0)	9,217 (84·1)	1,740 (15·9)
60-69	10,975 (39·8)	16,585 (60·2)	10,103 (60·9)	6,482 (39·1)	12,274 (74·0)	4,311 (26·0)
70-79	9,302 (37·4)	15,576 (62·6)	9,245 (59·4)	6,331 (40·6)	11,207 (72·0)	4,369 (28·0)
80+	4,309 (46·1)	5,036 (53·9)	3,422 (68·0)	1,614 (32·0)	3,983 (79·1)	1,053 (20·9)
**Deprivation n (%)**						
1 (most deprived)	10,198 (48·4)	10,856 (51·6)	8,174 (75·3)	2,682 (24·7)	9,269 (85·4)	1,587 (14·6)
2	9,986 (46·9)	11,305 (53·1)	7,857 (69·5)	3,448 (30·5)	9,021 (79·8)	2,284 (20·2)
3	8,683 (42·2)	11,899 (57·8)	7,673 (64·5)	4,226 (35·5)	9,017 (75·8)	2,882 (24·2)
4	7,875 (42·3)	10,730 (57·7)	6,708 (62·5)	4,022 (37·5)	8,068 (75·2)	2,662 (24·8)
5 (least deprived)	7,322 (41·5)	10,319 (58·5)	6,181 (59·9)	4,138 (40·1)	7,636 (74·0)	2,683 (26·0)
**Ethnicity[b] n (%)** (available for attenders only)						
White			24,117 (62·7)	14,322 (37·3)	28,993 (75·4)	9,446 (24·6)
Asian			5,157 (75·8)	1,647 (24·2)	5,778 (84·9)	1,026 (15·1)
Black			3,175 (73·9)	1,123 (26·1)	3,608 (83·9)	690 (16·1)
Other			1,457 (74·1)	510 (25·9)	1,650 (83·9)	317 (16·1)
**Employment n (%)** (available for attenders only)						
Employed			9,652 (72·8)	3,609 (27·2)	11,067 (83·5)	2,194 (16·5)
Retired			14,153 (59·3)	9,716 (40·7)	17,138 (71·8)	6,731 (28·2)
other			3,390 (74·6)	1,152 (25·4)	3,823 (84·2)	719 (15·8)
**Disability n (%)** (available for attenders only)						
no			25,080 (64·8)	13,620 (35·2)	29,713 (76·8)	8,987 (23·2)
yes			6,154 (68·2)	2,866 (31·8)	7,176 (79·6)	1,844 (20·4)

Supplemental Table S2 continuted: Participation by patient characteristics – descriptive summary

	**uptake full cohort n=99,473**		**60% attendance attenders n=55,275**		**completion attenders n=55,275**	
	no	yes	no	yes	no	yes
**Overall n (%)**	44,198 (44·4)	55,275 (55·6)	36,713 (66·4)	18,562 (33·6)	43,148 (78·1)	12,127 (21·9)
**Smoking n (%)** (available for attenders only)						
smoker			2,886 (79·7)	734 (20·3)	3,147 (86·9)	473 (13·1)
ex-smoker			83 (68·0)	39 (32·0)	106 (86·9)	16 (13·1)
non-smoker			24,998 (63·0)	14,703 (37·0)	30,081 (75·8)	9,620 (24·2)
**HbA1c (% (mmol/mol))**	**at referral**		**at initial assessment**		**at initial assessment**	
n median (IQR)	33,330 6.1 (43) (0.3 (3))	49,088 6.1 (43) (0.3 (3))	25,044 6.0 (42) (0.5 (5))	13,256 6.0 (42) (0.4 (4))	29,473 6.0 (42) (0.5 (5))	8,827 6.0 (42) (0.4 (4))
**FPG (mmol/l)**	**at referral**		**at initial assessment**		**at initial assessment**	
n median (IQR)	5,995 5·7 (0·7)	6,845 5·8 (0·6)	29 5·9 (0·6)	10 6·2 (0·7)	33 5·9 (0·6)	6 6·3 (0·8)
**Blood glucose category n (%)**	**at referral**		**at initial assessment**		**at initial assessment**	
normal	1,051 (60·6)	684 (39·4)	11,950 (66·9)	5,909 (33·1)	13,983 (78·3)	3,876 (21·7)
NDH	34,108 (39·8)	51,695 (60·3)	11,209 (61·8)	6,925 (38·2)	13,443 (74·1)	4,691 (25·9)
T2DM	318 (70·4)	134 (29·7)	1,912 (81·6)	432 (18·4)	2,078 (88·7)	266 (11·3)
**Weight (kg)**	**at referral**		**at initial assessment**		**at initial assessment**	
n median IQR			30,245 82·3 (24·4)	15,995 80·8 (23·4)	35,695 82·3 (24·2)	10,275 80·0 (23·2)
**BMI n (%)**	**at referral**		**at initial assessment**		**at initial assessment**	
underweight or healthy weight			4,920 (64·2)	2,745 (35·8)	5,803 (75·7)	1,862 (24·3)
overweight			10,606 (63·3)	6,136 (36·7)	12,697 (75·8)	4,045 (24·2)
obese			14,284 (67·2)	6,969 (32·8)	16,994 (80·0)	4,259 (20·0)

[a] Levels of missing data can be inferred, or see Table 8 for details

[b] ‘Asian’ comprises those reporting Indian, Pakistani, Bangladeshi, Chinese, or ‘other Asian’ ethnicity; ‘black’ comprises those reporting Caribbean, African or ‘other black’ ethnicity; ‘other’ comprises those reporting any other non-white ethnicity, including ‘mixed - ’ groups.

Supplemental Table S3: Participation by service characteristics – descriptive summary

	**uptake full cohort n=99,473**		**60% attendance attenders n=55,275**		**completion attenders n=55,275**	
	no	yes	no	yes	no	yes
**Overall n (%)**	44,198 (44·4)	55,275 (55·6)	36,713 (66·4)	18,562 (33·6)	43,148 (78·1)	12,127 (21·9)
**Provider n (%)**						
A	16,104 (40·6)	23,586 (59·4)	14,626 (62·0)	8,960 (38·0)	17,221 (73·0)	6,365 (27·0)
B	3,990 (37·4)	6,667 (62·6)	3,903 (58·5)	2,764 (41·5)	5,273 (79·1)	1,394 (20·9)
C	7,062 (60·5)	13,882 (66·3)	10,434 (75·2)	3,448 (24·8)	11,287 (81·3)	2,595 (18·7)
D	17,042 (60·5)	11,140 (39·5)	7,750 (69·6)	3,390 (30·4)	9,367 (84·1)	1,773 (15·9)
**Referral source n (%)**						
consultation (GP/ health check)	37,877 (53·3)	33,232 (46·7)	21,701 (65·3)	11,531 (34·7)	26,078 (78·5)	7,154 (21·5)
letter (self-referral after advice)	6,298 (22·2)	22,020 (77·8)	14,996 (68·1)	7,024 (31·9)	17,054 (77·4)	4,966 (22·6)
other sources	23 (50·0)	23 (50·0)	16 (69·6)	7 (30·4)	16 (69·6)	7 (30·4)
**Out-of-hours provision n (%)** (available for attenders only)						
none			30,960 (66·7)	15,440 (33·3)	36,210 (78·0)	10,190 (22·0)
some			5,753 (64·8)	3,122 (35·2)	6,938 (78·2)	1,937 (21·8)

Detail of coverage intervals used to illustrate variation in outcomes across sites

We followed the procedure described by Bartholomew et al 2008 to illustrate variation by site in multi-level models.(1,p335-336) Model variance estimates for random intercept terms (for CCG nested in STP) were examined In order to illustrate the variation in predicted outcomes due to variation between sites, after adjustment for patient and service characteristics. Together with the model assumptions,[1] these were used to calculate coverage intervals giving the predicted probability of each outcome across the middle 95% of sites, for a reference group with 'typical' values for individual and service characteristics. Typical characteristics were chosen to be the modal value for gender, disability, employment, smoking, referral source and provider, and values close to the median for other covariates, representing a white, 65 year old, retired, non-smoking woman in the middle deprivation group, reporting no disability, with HbA1c of 6.1% (43mmol/mol), weight 76kg, WEMWBS score 54, referred via consultation to a six month old service with provider A. As noted in the Discussion, many of these modal and median characteristics are associated with better than average outcomes, so that the overall proportion achieving 60% attendance lies below the 95% coverage interval for this reference group, while that for completion lies towards the lower bound of the corresponding interval.

Detail of missing data and multiple imputation

Multiple imputation was used to reduce bias due to missingness among covariate values of up to 30%, see Table 8 for details. Fully conditional specification (FCS) was used due to the large number of variables of mixed continuous and categorical types to be used.(2; 3) Imputation was performed separately for non-attenders and attenders, since variables collected at initial assessment were plausibly missing not at random

[1] STP and CCG effects are assumed to be independently normally distributed with mean 0 and variance $\hat{\sigma}^2_{STP}$ and $\hat{\sigma}^2_{CCG}$ respectively; it is therefore expected that around 95% of sites will have combined site effects $(u_{STP} + u_{CCG})$ within $\pm 1.96\sqrt{\hat{\sigma}^2_{STP} + \hat{\sigma}^2_{CCG}}$ of 0.

among non-attenders (i.e. differently distributed among non-attenders compared with attenders, conditional on observed data).(4) Therefore, these were neither imputed nor included in analysis of uptake among all referrals, but were added to imputation and analysis models for retention and completion among attenders.(5) This approach ensured that for each analysis, all covariates, outcomes and auxiliary variables were included in the FCS for the corresponding subsample.(6)

Supplemental Table S4: Variables used in imputation models

	% missing (all referrals n=99,473)	% missing (attenders n=55,275)
Substantive covariates		
Age	Complete	
Deprivation	Complete	
Provider	Complete	
Referral source	Complete	
STP	Complete	
Gender	1	<1
HbA1c at referral	15	10
Out-of-hours provision		Complete
Ethnicity		7
Employment		25
Disability		14
Smoking		21
HbA1c at initial assessment		30
Weight at initial assessment		16
WEMWBS at initial assessment		27
Outcomes		
Uptake	Complete	
Retention to 60%		Complete
Completion		Complete
Auxiliary variables		
Service maturity	Complete	
Neighbourhood proportion BAME residents	Complete	
Height	39	4

After small groups had been excluded,[2] complete covariates age, deprivation, provider, referral source and STP[3] and the outcome uptake were included in the FCS for non-attenders, with parameters estimated from among all referrals. Complete covariate out-of-hours delivery and complete outcomes retention and completion were added to the FCS for attenders. Participation outcomes were completely observed, assuming that missing attendance data represented non-attendance given funding incentives to providers to record attendance. Service maturity (months since establishment to referral; complete), neighbourhood proportion of black and minority ethnic (BAME) residents obtained from census data 2011 (complete) and height (incomplete) [4] were included as auxiliary variables for imputation among both subsamples.

Multinomial logistic imputation models were used for categorical variables, with augmented regression to overcome perfect prediction of categorical variables (sex, ethnicity, employment, disability and smoking).(7) Linear regression models were used for log-transformed weight and log-transformed neighbourhood proportion BAME residents, from which untransformed values were passively imputed, and

[2] Those reporting gender as 'other' (n=31), those referred via methods other than consultation or letter (n=46), those transferred to wave 3 services (n=73) and those with age or deprivation missing (n=317); n=467 in total giving a reduced cohort of n=99,006.
[3] STP was omitted from the imputation models for ethnicity and gender due to perfect prediction for many individuals.
[4] Although height was collected at initial assessment it was assumed to be missing at random and imputed for all referrals.

for height. Predictive mean matching was used for Hba1c and WEMWBS score. Clustering by site was accounted for by including STP as a covariate in the imputation models (subgroups defined by CCG were too small to include CCG as a covariate).

Ten imputations were performed using a burn-in of 25, chosen after examination of trace plots to check for convergence. Monte Carlo error (MCE) for coefficient estimates was less than 10% of the corresponding estimated standard errors; MCE estimates for test statistics were below 0.1.(2)

A complete case analysis (CCA) gave similar results in most cases, but with quite different estimates of provider effects for all three milestones due to wide variation in levels of missingness across providers, with participation lower among cases with missing covariates· Thus, the extent of variation across providers would be underestimated for all three milestones using a CCA, and furthermore the association with out-of-hours provision would be somewhat attenuated.

References

1. Bartholomew DJ, Steele F, Moustaki I. *Analysis of multivariate social science data*. CRC press, 2008
2. White IR, Royston P, Wood AM. Multiple imputation using chained equations: Issues and guidance for practice. Statistics in medicine 2011;30:377-399
3. Audigier V, White IR, Jolani S, Debray T, Quartagno M, Carpenter J, van Buuren S, Resche-Rigon M. Multiple imputation for multilevel data with continuous and binary variables. arXiv preprint arXiv:170200971 2017;
4. Rubin DB. *Multiple imputation for nonresponse in surveys*. John Wiley & Sons, 2004
5. Allison PD. Missing Data. Thousand Oaks, California, 2002
6. Kontopantelis E, White IR, Sperrin M, Buchan I. Outcome-sensitive multiple imputation: a simulation study. BMC Med Res Methodol 2017;17:2-2
7. White IR, Daniel R, Royston P. Avoiding bias due to perfect prediction in multiple imputation of incomplete categorical variables. Computational statistics & data analysis 2010;54:2267-2275
